# Oncolytic virotherapy for malignant glioma: translating laboratory insights into clinical practice

**DOI:** 10.3389/fonc.2013.00032

**Published:** 2013-02-25

**Authors:** Brenda Auffinger, Atique U. Ahmed, Maciej S. Lesniak

**Affiliations:** The Brain Tumor Center, The University of ChicagoChicago, IL, USA

**Keywords:** oncolytic virotherapy, malignant glioma, cancer stem cells, immunomodulation, challenges

## Abstract

Glioblastoma multiforme, one of the most common and aggressive brain tumors in adults, is highly resistant to currently available therapies and often recurs. Due to its poor prognosis and difficult management, there is an urgent need for the development and translation of new anti-glioma therapeutic approaches into the clinic. In this context, oncolytic virotherapy arises as an exciting treatment option for glioma patients. These natural or genetically engineered viruses are able to effectively infect cancer cells, inducing a specific anti-tumor cytotoxic effect. In addition, some viruses have been redesigned to modulate glioma microenvironment, to express cytokines to boost a systemic anti-glioma immune response and to incorporate angiostatic genes to decrease glioma vasculature. Although recent clinical trials have confirmed the safety of oncolytic virotherapies in the brain, their moderate clinical efficacy has not yet matched the encouraging preclinical laboratory results. In this review, we will discuss the leading anti-glioma virotherapy approaches that are presently under preclinical and clinical evaluation. We will also review different delivery methods, *in vivo* virus behavior, fate, replication, intratumoral spread, activation of anti-tumor immune response, and targeting of glioma stem cells. We will focus on the advantages and limitations of each therapeutic approach and how to overcome these hurdles to effectively translate exciting laboratory results into promising clinical trials.

## Introduction

Malignant glioma is a highly invasive brain tumor characterized by short survival and poor response to chemotherapeutic agents (Grossman et al., 2010). Biological barriers and physiological aspects responsible for hampering a broad intratumoral drug distribution and efficient tumor destruction can explain this poor therapeutic efficacy. Important examples are the presence of an ultra-selective blood-brain barrier, glioma heterogeneity, existence of drug-resistant glioma stem cells, and low anti-tumor immunogenicity (Wen and Kesari, 2008; Stupp and Roila, 2009). In this scenario, oncolytic virotherapy arises as a promising approach to restrain glioma-related deaths. This new treatment option is based on the rationale of using conditionally replicative viruses to either selectively infect and kill glioma cells, sparing non-neoplastic tissues (Parker et al., 2009), as well as transfer genetic materials with anti-cancer activity to cancer cells through viral vectors (Fueyo et al., 1999; Bansal and Engelhard, 2000). Oncolytic viruses induce an anti-tumor therapeutic effect through a subtle equilibrium between anti-viral and anti-tumor immune responses (Fulci et al., 2006). In order to achieve efficient oncolytic activity a viral vector must obey three main principles: (1) selectively target the neoplastic tissue while presenting minimal local and systemic toxicity, (2) remain active despite inducing host anti-viral immune response, and (3) reach all tumor foci beyond the tumor resection border. In addition to this, it must be safe for human administration, and should demonstrate potent anti-tumor activity either alone or combined with conventional therapies, such as surgical resection, chemotherapy, and radiotherapy (Dey et al., 2011). However, several limitations are still present for an adequate translation of oncolytic virotherapy into the clinics. One of the major obstacles is the absence of an acceptable vector system that could be administered to the patient in a minimally invasive fashion and would transduce and lyse most of the tumor with low toxicity to normal tissues (Hunt and Vorburger, 2002). Other therapeutic hurdles comprise anti-vector immune response, which limits a repeated viral administration; lack of long lasting anti-tumor immune response, which allows glioma recurrence; and unreliability of current animal models, where therapeutic effectiveness usually does not translate to success in human trials (Donsante et al., 2007). In this review, we will discuss the new anti-glioma virotherapies that might be used alone or in conjunction with conventional therapeutic approaches and have the potential to offer an advantage over currently employed therapeutic regimens. We will also outline the advantages and pitfalls of such therapeutic approach, as well as discuss feasible alternatives to overcome these limitations and effectively translate anti-glioma oncolytic virotherapies from bench to bedside.

## Targeted anti-glioma virotherapy

Two classes of viruses are currently employed in anti-glioma targeted virotherapy: replication- defective and replication- competent ones. Replication-defective viruses are mainly used as vectors for suicide gene delivery, while replication-competent viruses exert their therapeutic effect through either direct lysis of tumor cells or modulation of glioma-related apoptotic pathways (Biederer et al., 2002). Replication-competent viruses can be genetically engineered into conditionally replicative viral vectors. In this case, viral replication is restricted to neoplastic cells and therapeutic genes carried by these vectors can be amplified at the tumor site. Another important difference is that replication-competent viruses show higher tumor transduction efficiency, characteristic that makes them important tools in both oncolytic viral therapy and gene therapy (Heise et al., 2000; Jiang et al., 2003; Lin and Nemunaitis, 2004). Here, we describe three widely used oncolytic virus systems that present an established anti-glioma activity and are presently under clinical trials: conditionally replicative adenoviruses (CRAd), oncolytic herpes simplex virus (oHSV) and reovirus.

### Conditionally replicative adenoviruses (CRAd)

Human adenovirus serotype 5 (Ad5) is a well-characterized platform for a wide variety of genetically modified oncolytic virus agents. It comprises a 36 kb DNA genome that allows for relatively easy manipulation. This fact opened new paths for designing vectors that present high specificity for tumor cells, limiting toxicity to non-neoplastic tissues. Three main modifications in the viral replication pathway allowed such tumor-specific tropism: (1) deletion of viral E1A or E1B genomic regions, which limited viral replication to cancer cells with specific dysfunctions in cell cycle checkpoint pathways (Khuri et al., 2000; Suzuki et al., 2001); (2) incorporation of tumor-specific promoters into the viral genome, which limited infection only to malignant cells expressing such particular promoter (Vandier et al., 2000; Kohno et al., 2004); and (3) improvement of transduction efficiency in tumor cells, which redirected the process of virion entry into target cells (van Beusechem et al., 2002; Fueyo et al., 2003; Dey et al., 2011). In addition, adenoviruses can also be useful tools in anti-glioma gene therapy, mainly through bearing a suicide cassette that induces the conversion of prodrugs into active drugs (HSV-tk system).

**Figure 1:**
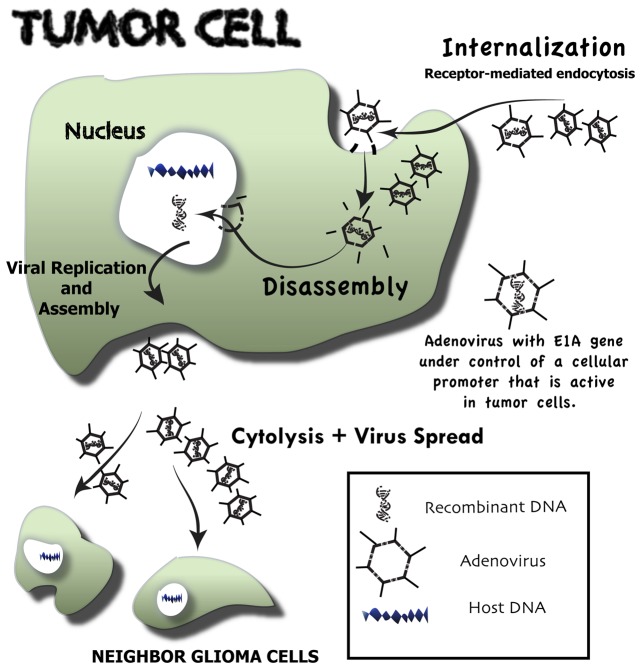
**Overview of the events that follow upon conditionally replicative adenovirus infection of malignant glioma cells—oncolytic virotherapy.** Neoplastic cells through a receptor-mediated endocytosis selectively internalize genetically engineered conditionally replicative adenoviruses (CRAds). These CRAds are tumor-selective because their E1A gene is under control of a cellular promoter that is active only in tumor cells. Following internalization, CRAds are engulfed by endosomes, where vectors are disassembled and viral DNA is released. Viral DNA is directly transported to the nucleus of the neoplastic host cell. There, it highjacks the host DNA machinery, which is redirected for viral DNA production. Vector assembly takes place in the cytosol, where viral capsid proteins are produced. Upon assembly, a great number of newly formed conditionally replicative adenoviruses cause tumor cell cytolysis and then migrate to other glioma cells, where the whole process is repeated.

**Figure 2:**
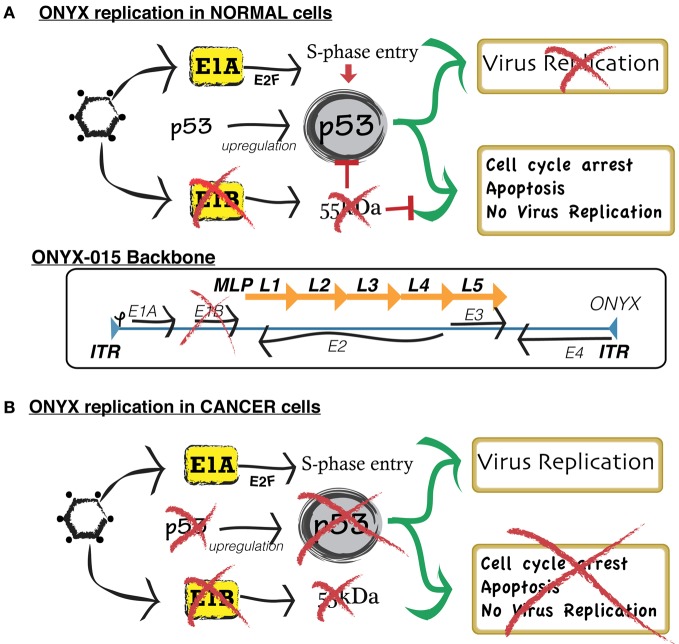
**Simplified scheme comparing the mechanism of replication of conditionally replicative ONYX in normal cells and neoplastic cells. (A)** In normal cells, wild type adenoviruses replicate by blocking the normal activity of p53, a gene that, by inducing cell apoptosis, defends the host cell from viral infection. In order to exploit such function for targeted cancer therapy, researchers developed ONYX-015. ONYX-015 is a genetically engineered oncolytic adenovirus that preferentially replicates in neoplastic cells. It contains an 827-bp DNA deletion in the E1B region of the viral genome, which is responsible for the production of a mutant E1B-p55 protein. In normal cells, ONYX-015 induces a p53 response, leading to cell arrest and apoptosis, therefore preventing vector replication and contamination of non-cancer cells. **(B)** Cancer cells present a disrupted p53 pathway. Therefore, they are unable to suppress viral replication.

Retinoblastoma protein (Rb) and protein 53 (p53) tumor suppressors are master cell cycle regulators. They hold cell cycle at G1/S regulation checkpoints for DNA damage recognition. As G1 to S transition are essential for adenovirus replication, E1A and E1B early viral genes control cell cycle by inhibiting Rb and p53, ensuring efficient DNA replication (Gomez-Manzano et al., 2004). Nevertheless, malignant gliomas present a defective p53 and Rb pathways, contributing to uncontrolled cell division and genome instability (Bischoff et al., 1996). Thus genetically engineered adenoviruses with either E1A or E1B deletion replicate preferentially in malignant cells with disrupted p53 and Rb proteins, but not in non-neoplastic cells (Figure 1). ONYX-015, an adenovirus made conditionally replicative by E1B-55k deletion, has shown *in vivo* therapeutic effects in glioblastoma xenografts (Figure 2) (Geoerger et al., 2002). Likewise, Ad5-Delta24 bears a partial E1A deletion, which has proven to be more effective than ONYX-015 in suppressing tumor growth in both intracranial and subcutaneous glioma xenografts (Fueyo et al., 2000). In addition to rendering E1A unable to bind Rb, Ad5-Delta24 induces topoisomerase I expression in malignant cells. As a consequence, it has its antitumor effect synergistically improved by irinotecan, a topoisomerase I inhibitor, in experimental murine models (Jiang et al., 2005). Based on promising preclinical results, ONYX-015 has reached clinical trials and, in 2004, Chiocca et al. (2004) reported a phase I trial conducted with intratumorally injected ONYX-015. In this study, 24 patients suffering from recurrent malignant glioma received ~10^10^ pfu (plaque-forming units) at 10 different sites around the tumor resection border. Results showed that the virus was well tolerated and no toxicity has been noted (Table 1).

**Table 1:** **Completed and ongoing clinical trials using oncolytic virotherapy as a therapeutic strategy against malignant glioma**.

**Virus type**	**Genetic alteration**	**Target**	**Study type**	**Delivery method**	**Status of clinical trial**	**Reference**
Ad: ONYX-015	E1B-55k deletion	Deficient p53 pathway	Phase I	Tumor bed post-resection	Completed	Chiocca et al., 2004
Ad: Ad5-Delta24RGD	Partial E1A deletion and received RGD motif	Disrupted Rb pathway	Phase I	Intratumoral	Recruiting patients	NCT00805376
Ad: Ad5-Delta24RGD	Partial E1A deletion and received RGD motif	Disrupted Rb pathway	Phase I/II	Intratumoral/CED	Recruiting patients	NCT01582516
Ad: AdV-tk	Thymidine kinase (tk) incorporation	Intracellular activation of prodrugs	Phase IB	Tumor bed post-resection	Completed	Chiocca et al., 2011
Ad: AdV-tk/GCV	Thymidine kinase (tk) incorporation	Intracellular activation of prodrugs	Phase I	Tumor bed post-resection	Completed	Sandmair et al., 2000
RV: RV-tk/GCV	Thymidine kinase (tk) incorporation	Intracellular activation of prodrugs	Phase I	Tumor bed post-resection	Completed	Sandmair et al., 2000
HSV-1: G207	γ_1_34.5 gene deletion and lacZ insertion in U_L_39	Protein phosphatase 1a and disrupted IFN pathway	Phase I	Intratumoral	Completed	Markert et al., 2000
HSV-1: G207	γ_1_34.5 gene deletion and lacZ insertion in U_L_39	Protein phosphatase 1a and disrupted IFN pathway	Phase IB	Intratumoral and tumor bed post-resection	Completed	Markert et al., 2009
RV: Reovirus Wild type	None	Activated Ras pathway	Phase I	Intratumoral	Completed	NCT00528684 (Forsyth et al., 2008)

Abbreviations *Ad* *adenovirus* *E1B* *adenovirus E1B protein* *p53* *protein 53* *Ad5* *human adenovirus serotype 5* *RGD* *arginyl-glycyl-aspartic acid motif* *E1A* *adenovirus early region 1A* *Rb* *retinoblastoma protein* *CED* *convection enhanced delivery* *AdV* *adenovirus* *tk* *thymidine kinase* *GCV* *ganciclovir* *RV* *reovirus* *IFN* *interferon* *Ras* *Ras pathway.*

Tumor-specific promoters can be incorporated into the adenoviral genome in order to produce conditionally replicative viral vectors. Following this rationale, Komata et al. (2001) engineered a viral vector expressing a constitutively active caspase-6 under hTERT promoter. HTERT is a well-known regulator of the telomerase enzyme, which is responsible for chromosomal stabilization and avoidance of senescence, rendering cells the capacity of unlimited divisions. This enzyme is present in neoplastic tissues, but is absent in normal brain. Based on this, such hTERT/rev-caspase-6 viral construction is able to specifically target and lyse hTERT-positive tumor cells. Promising *in vivo* results have shown tumor regression in subcutaneous nude mice xenografts. Another example of promoter incorporation in viral vectors is the construction of a hypoxia/HIF-dependent replicative adenovirus (HYPR-Ad), which is able to target hypoxic malignant glioma cells (Post and Van Meir, 2003). Such targeted mechanism happens through a hypoxia-dependent E1A promoter expression, resulting in conditional cytolysis of hypoxic cells, sparing the normal tissue. Similarly, CRAd-survivin constructs comprise oncolytic adenoviruses in which replication is controlled by survivin promoter, an apoptosis inhibitor (Van Houdt et al., 2006). Adenoviral fiber gene modifications that enhance glioma-specific viral targeting have been incorporated in these survivin-controlled CRAds and have shown promising results both *in vitro* and *in vivo* (Ulasov et al., 2007b,c; Nandi et al., 2008; Sonabend et al., 2009). Examples involve the addition of an RGD-modified fiber (Zhu et al., 2005), a poly-lysine motif (CRAd-S-pk7) (Ulasov et al., 2007c) and a chimeric fiber Ad3 knob (Ulasov et al., 2007b). Despite their promising pre-clinical results, no clinical trials using these constructs are currently available.

New viral constructions that facilitate interactions between viral proteins and particular cell surface receptors lead to an increased viral transduction in malignant cells. This has been mainly achieved by deletion of specific portions of the viral genome (Gomez-Manzano et al., 2004; Ulasov et al., 2008) or by addition of exogenous promoters (Ulasov et al., 2007a). For instance, Ad5-Delta24RGD is an engineered adenoviral vector that, by receiving an arginyl-glycyl-aspartic acid (RGD) motif, is able to interact with αvβ_3_ and αvβ_5_ integrins, which present ample expression in neoplastic cells (Suzuki et al., 2001). This coxsackievirus and adenovirus receptor (CAR) independent transduction showed a strong oncolytic effect in a broad panel of primary gliomas, together with complete tumor regression and long-term survival in an *in vivo* glioma xenograft model (Lamfers et al., 2002). In addition, Wang et al. demonstrated that bFGF2 (basic fibroblast growth factor 2) could be used as a targeting ligand in order to maintain adenoviral infection specific to glioma cells. They showed that such viral construct was especially effective in gliomas that expressed low CAR (Wang et al., 2005). Presently, a phase I clinical trial using Delta-24-RGD is recruiting recurrent malignant glioma patients (NCT00805376). This study intends to measure the maximum tolerated dose of this double modified (DNATrix) conditionally replication-competent adenovirus by injecting ~10^10^ pfu at the tumor site.

In addition to oncolytic viruses, replication incompetent viruses also present important anti-tumor responses. Herpes simplex type 1 thymidine kinase (HSV-tk) is a suicide cassette responsible for the conversion of the inactive prodrug ganciclovir (GCV) into an active toxic metabolite named GCV-triphosphate (Gomez-Manzano et al., 2012). It can be incorporated into the adenoviral genome in order to enhance killing of adenovirus-infected tumor cells as well as non-infected neighboring neoplastic cells through a “bystander effect” (Chen et al., 1995). Due to its impressive *in vitro* and *in vivo* results, such therapeutic approach reached clinical trials (Chen et al., 1995; Sandmair et al., 2000). Recently, Chiocca et al. (2011) reported a phase IB trial in which newly diagnosed glioblastoma patients received a single injection of adenovirus-tk (AdV-tk) at the tumor resection site followed by 14 days of the oral drug vancyclovir. This study showed that the AdV-mediated HSV-tk system was well tolerated by patients with no significant added toxicity. Another randomized, controlled clinical trial developed in Finland studied 36 patients with primary or recurrent malignant glioma (Immonen et al., 2004). All 17 patients from the randomized group were treated with AdV-HSV-tk gene therapy (3 × 10^10^ pfu) at the tumor resection bed, followed by intravenously administered GCV, twice a day for 14 days. The control group formed by 19 patients was treated with standard care radical surgical resection followed by radiotherapy. All patients treated with AdV-HSV-tk presented a significantly increased median survival when compared to the control group (62.4 vs. 37.7 weeks). The treatment was well tolerated, with no adverse effects. Six patients presented increased anti-adenovirus antibody titer. In parallel to this, Sandmair et al. (2000) reported on a phase I clinical trial where malignant glioma patients were treated either with replication-defective AdV bearing HSV-tk/GCV or reovirus-mediated HSV-tk/GCV at the tumor resection margin. The results of this trial showed that patients that received AdV-HSV-tk presented a significantly higher median survival (~15 months) when compared to the other group (~7.4 months). These results suggest that replication-defective adenoviruses may be a better choice over reoviruses for suicide gene therapy. However, recent reports suggest that enhanced anti-tumor responses would be achieved upon the combination of replication-defective and oncolytic virotherapies. Therefore, it would be interesting to invest in additional studies combining both targeted approaches in preclinical and clinical models.

### Oncolytic herpes simplex virus (oHSV)

Herpes simplex virus-1 (HSV-1) is a DNA virus containing a large (>150 kb), fully sequenced and well-characterized genome (Chou et al., 1990). This neurotropic virus presents a number of features that makes it an attractive option for brain cancer therapy. It is able to infect a large panel of malignant cells, requiring relatively few replication copies for appropriate cell destruction. Its ability to remain as an episome prevents insertional mutagenesis into the host genome and allows the easy activity of antiviral drugs, effectively controlling viral replication. Infected hosts present an enhanced anti-tumor immune response and circulating anti-HSV1 antibodies do not affect viral spread to other tumor cells (Todo, 2012). Moreover, the currently available anti-glioma oncolytic HSV-1 patents mostly rely on three types of viral genome modifications: (1) removal of genes that are not essential for viral replication, followed by insertion of therapeutic transgenes in the viral backbone, (2) induction of specific anti-tumor immunity by the insertion of costimulatory molecules, and (3) construction of oHSV-1 viruses armed with immunostimulatory agents.

Due to its large genome, HSV-1 backbone tolerates the removal of genes that are not crucial for viral replication and the insertion of either large or multiple therapeutic genes, opening innumerous opportunities for anti-glioma gene therapy. As such, G207, the first oHSV-1 used in anti-glioma therapy, presents a deletion of the γ_1_34.5 gene in its both alleles, together with a lacZ insertion into the U_L_39 locus, blocking its function (Mineta et al., 1995). These two mutations are responsible for restraining G207 replication to neoplastic cells and rendering it vulnerable to standard anti-viral therapies. After extensive *in vivo* safety evaluations, a phase I clinical trial evaluated the therapeutic efficacy of G207 in recurrent malignant glioma patients (Markert et al., 2000). Participants were treated with intratumoral injections of ~10^9^ pfu. As a result, no adverse effects were noticed and tumor regression was detected by serial MRI evaluations. Two patients survived more than 5 years. These exciting outcomes encouraged a phase IB trial (Markert et al., 2009), in which recurrent glioma patients received G207 conditionally replicative viruses either intratumorally or at the resection site. RT-PCR of biopsied samples indicated *in situ* viral replication. Although no HSV-1 related encephalitis was observed, progression free survival by MRI was only 3 months, with a median survival of 6.6 months.

Preclinical studies using immunocompetent animals showed that G207 was capable of inducing a systemic anti-tumor immunity in experimental brain tumor models (Todo et al., 1999). This result has proven that a successful oHSV-1 therapy depends not only on the extent of viral replication, but also on induction of host-related anti-tumor response. Based on this, generation of specific anti-tumor immunity by the insertion of costimulatory molecules in the viral genome showed itself as an attractive approach. The *in vivo* efficacy of a genetically engineered replication-defective HSV-1 (dvB7Ig) expressing a potent costimulatory molecule, B7-1, has been tested in conjunction with G207 (Galea-Lauri et al., 1996; Todo et al., 2001a). This dvB7Ig/G207 system was capable of inhibiting tumor growth in subcutaneous models and prolonging mouse survival in intracranial models, conferring tumor-specific protective immunity on cured mice. In addition, further studies showed that such anti-tumor effect was dependent on CD8^+^ T cells, but not CD4^+^ T cells.

Oncolytic HSV-1 viruses armed with immunostimulatory agents are considered the “next generation” of HSV-related anti-neoplastic therapies. The viruses that have such constructs incorporated in their genomes present many attractive advantages over the so-called “non-armed” ones, such as *in vivo* amplified gene delivery with high levels of transgene expression and continuous generation of high-titer vectors. For instance, M002 and NV1042 engineered HSV-1 viruses expressing IL-12 displayed improved *in vivo* therapeutic effectiveness in glioma preclinical models (Parker et al., 2000; Hellums et al., 2005). Likewise, the use of a γ_1_34.5 replication-deficient HSV-1 holding an IL-4 gene insertion, exhibited noteworthy tumor regression in intracranial models with prolonged mouse survival. Cured mice that were treated with NV1042 presented a high rate of rejection of rechallenged tumors (Andreansky et al., 1998). Moreover, it was recently reported that a combination of three armed viruses displayed stronger anti-tumor activity than any armed virus alone or the combination of two viruses (Todo, 2012). This finding opens a new research avenue, where different oncolytic viruses can be combined in order to achieve potent anti-tumor efficacy.

### Reovirus

Reovirus is a double-stranded RNA virus that can easily transduce most mammalian cells (Kim et al., 2007). Literature reports have indicated that reovirus permissiveness correlates with the activation status of Ras signaling pathways in the host cell (Strong et al., 1998). In humans, reovirus commonly causes only a mild disease due to limited viral gene translation and ineffective infection of normal cells. This mainly happens because non-neoplastic cells present an inactivated Ras pathway, which leads to activation of protein kinase R (PKR) and phosphorylation of eukaryotic initiation factor 2, limiting reovirus infectivity (Strong et al., 1998). However, in tumor cells the oncogenic Ras activated pathway prevents PKR activation, allowing an effective viral gene translation, which results in satisfactory reovirus infection and cytolysis (Quinlan et al., 2008). Thus, reovirus's natural preference for cells with unrestricted Ras pathway activity together with its non-pathogenic profile in humans has made it an interesting candidate for brain tumor oncolytic virotherapy. Experimental glioma models have proven *in vitro* and *in vivo* reovirus therapeutic effectiveness as well as its natural limitation to tumor cells (Coffey et al., 1998; Kottke et al., 2010). Although serious adverse reactions have been reported upon reovirus inoculation in severe combined immunodeficiency mice (SCID), such toxicity has not been observed in other models (Coffey et al., 1998; Yang et al., 2003). After extensive assurance of reovirus safety in primates and non-SCID models, such virus was finally approved for clinical trials (Yang et al., 2004). Two phase I clinical studies wherein escalating doses of therapeutic reovirus (Reolysin®) were intratumorally injected in recurrent glioma patients have been concluded (NCT00528684) (Forsyth et al., 2008). In the first study, 12 patients received ~10^9^ pfu of reoviruses in three intratumoral injection sites. As a result, no virus-related adverse events were reported and no dose-limiting toxicity was observed. One out of twelve patients remained disease-free for more than 6 years. In the second study, 18 patients were intratumorally treated with ~10^9^ pfu of reoviruses by convection enhanced delivery (CED). One partial therapeutic response was observed and three patients remained stable. No dose-limiting toxicity was reported. These studies demonstrated that replication competent reoviruses could be effectively used as a therapeutic strategy against malignant glioma. Such outcomes open doors for other clinical investigations, in which the efficacy of reovirus combined with conventional therapies, could be further evaluated in humans.

## Targeting cancer stem cells

The cancer stem cell (CSCs) hypothesis proposes that neoplastic clones are exclusively maintained by a small fraction of tumor-initiating cells that, similarly to normal stem cells, possess stem-like properties such as relative quiescence, multipotency, and self-renewal (Stupp et al., 2005). When isolated and serially implanted in immunodeficient mice, these cells have the ability to recapitulate the original tumor, reproducing all of its complexities and primary features (Hamburger and Salmon, 1977). Evidence indicates that these progenitor-like cells are responsible for tumor infiltration, progression, and metastasis (Hermann et al., 2007; Stupp and Hegi, 2007). In addition to this, expression of drug transporters and enhanced DNA repair mechanisms render these cells resistant to conventional cytotoxic treatments, which usually target chemo- and radiosensitive rapid dividing cells of the tumor bulk and neglect the resistant CSCs (Dean et al., 2005). Accordingly, it is believed that surviving tumor-initiating cells are responsible for glioma recurrence post initial therapy (Bao et al., 2006). Therefore, targeting specific CSC properties with oncolytic virotherapy is an attractive approach to avoid glioma relapse. Two specific modifications in the viral backbone enhance targeting of CSCs: (1) insertion of CSC promoters in the viral genome, enabling a gene therapy that targets both CSCs and non-CSCs, and (2) modification of the viral capsid in order to improve viral transduction and enrich CSC targeting.

Promoters that are present in both malignant cells and CSCs can be introduced into the genome of conditionally cytotoxic viruses, enhancing CSC virotherapy. Following this concept, Zhang et al. engineered a telomerase-specific conditionally replicative adenovirus vector that incorporated a TNF-related apoptosis inducing ligand (Ad/TRAIL-E1A). Mice bearing radioresistant stem cell-enriched esophageal cancer xenografts treated with such vector presented important suppression of tumor growth and longer survival (Zhang et al., 2008). In addition to adenoviruses, HSV-1 viruses have also been employed in oncolytic virotherapy that target CSCs. Wakimoto et al. used an oHSV with ICP6, γ_1_34.5, and α47 deletions (G47Delta) for the treatment of intracranially implanted stem cell-enriched glioma xenografts. Results showed that G47Delta was able to selectively kill and suppress CSC growth as well as significantly prolong survival of nude mice (Wakimoto et al., 2009). G47Delta has also been tested in combination with chemotherapeutic agents like etoposide (Alonso et al., 2012) and temozolomide (Cheema et al., 2011). As a result, both preclinical studies displayed increased apoptosis of glioma cells and extended survival.

The rate of viral transduction in tumor-initiating cells can be enhanced through specific modifications on the viral capsid. In order to enrich CSC targeting, Jiang H et al. engineered an oncolytic adenovirus (Delta-24-RGD) with selective replication in tumor cells with a defective p16INK4/Rb pathway. Such construct showed exciting therapeutic outcomes in glioma intracranial models. Upon *in vivo* challenging, Delta-24-RGD viruses displayed efficient replication and induced death of both glioma stem cells and non-CSCs (Jiang et al., 2007). Based on such results, two phase I clinical trials using intratumoral injections of Delta-24-RGD in patients with recurrent malignant glioma were recently initiated (NCT00805376) (NCT01582516) (Alonso et al., 2012).

Although preclinical studies indicate that oncolytic virotherapy is able to successfully target those CSCs that escaped conventional treatments, some big challenges are still on the way for the development of an ideal vector. An important hurdle yet to be overcome is the shortage of promoters that are known to be exclusively present in CSCs. Due to the marked similarity between CSCs and normal stem cells, a non-specific promoter that is responsible for inducing apoptosis in CSCs will also cause death of their normal counterparts. For these same reasons, optimizing CSC-specific viral transduction is still a challenge. The lack of surface receptors that are specific for CSCs hinders current efforts for an efficient modification of viral fiber knobs that would solely bind to CSCs. Therefore, the discovery and characterization of markers that are specific for CSCs and that could enhance their targeted therapy are still in urgent need.

## Challenges in developing efficient anti-glioma targeted virotherapies

Since its introduction, remarkable progress was achieved in the oncolytic virotherapy research field. The development of conditionally replicative oncolytic viruses that are able to function both as tumor-specific apoptosis inducers and gene delivery vehicles has revolutionized glioblastoma therapeutics. Many of these viruses showed significant efficacy in preclinical models and established good safety profiles in phase I clinical trials. Despite such promising results, this therapeutic approach still faces serious limitations. First, the absence of a vector system with minimally invasive administration and high effectiveness is still a big challenge for the wide incorporation of this approach into the clinics. Second, physical barriers within the tumor microenvironment that preclude efficient viral replication and spread through the tumor interstitium are still responsible for decreased rates of neoplastic cell infection and inefficient tumor eradication. Third, a strong host anti-vector immune response post-virus administration, which inhibits efficient virus transduction and replication in tumor cells, together with the lack of a long lasting anti-tumor immune response are also important barriers to overcome. Last, inaccurate preclinical animal models that are usually not representative of the heterogeneous nature of the human glioma are known to deliver exciting therapeutic results that are mostly non-reproducible in clinical trials. In this section, we will discuss various limitations that hamper an effective translation of anti-glioma targeted virotherapies from a preclinical setting to the clinics. We will also survey some possible alternatives to overcome such obstacles.

### Delivery limitations

Malignant gliomas pose a unique therapeutic challenge. As complete surgical removal of the tumor is not feasible due to the infiltrative nature of glioma and dissemination away from the primary tumor site, chemotherapy and radiotherapy are usually combined in an attempt to kill remaining neoplastic cells. However, conventional cancer treatments do not succeed in killing chemo- and radio-resistant CSCs, which often lead to tumor recurrence. In addition, off-target effects of conventional therapies frequently result in high cytotoxicity to normal tissues and adverse side effects. The therapeutic efficacy of systemic chemotherapeutic agents is also significantly limited due to the presence of a highly selective BBB (Neuwelt, 1980). In this scenario, oncolytic virotherapy emerges as an important tool for targeted anti-glioma therapy. The systemic delivery of oncolytic viruses into tumors has been long studied. However, it has proven to be inefficient because of liver sequestration of intravenously administered viruses and poor central nervous system penetration (Streck et al., 2006). As a consequence, most oncolytic viruses that reached clinical trials are locally delivered during craniotomy, either intratumorally or on the tumor margins post-resection. However, the poor penetration of locally injected oncolytic vectors in the brain tissue poses an important limitation for this approach (Lang et al., 2003). To overcome this issue, researchers have developed a new delivery method named convention enhanced delivery (CED) (Bobo et al., 1994). Such approach relies on intracranial delivery of viral vectors through continuous infusion via catheters. Its advantage over local injections is that it enables effective distribution of bigger volumes over large tumor areas (Mardor et al., 2005). CED has recently reached phase I/II clinical trials. Its main goal is to enhance transduction efficiency of viral vectors, such as reoviruses (NCT00528684) (Forsyth et al., 2008) and replication-incompetent semliki forest viruses (Ren et al., 2003), in recurrent glioma patients. So far they have proven moderate anti-glioma efficacy, but further safety studies are still necessary.

Although intracranial virus delivery through CED demonstrated a relative therapeutic effect, its full clinical success is still threatened by a strong host-mediated anti-viral immune response. To tackle this issue, stem cell-based delivery of oncolytic virotherapy has emerged as an alternative to shield therapeutic viruses from the innate immune system and, at the same time, to efficiently increase viral distribution to distant tumor areas. In order to fulfill such goals, carrier stem cells should (1) maintain their inherent tumor-tropic properties post-viral transduction, (2) be permissive to viral infection as well as support viral replication, and (3) effectively hide viruses from host immunosurveillance (Raykov et al., 2004). Recent *in vivo* studies demonstrated that well-characterized lacZ and CD-positive neural stem cells (NSCs), when systemically injected in nude mice bearing intracranial or subcutaneous flank tumors, localized to multiple tumor sites with almost no accumulation in non-neoplastic tissues (Brown et al., 2003). Moreover, delivery of CRAd by both MSCs (mesenchymal stem cells) (Sonabend et al., 2008) and NSCs (Tyler et al., 2009) to distant gliomas was significantly more efficient than virus injection alone, with enhanced intratumoral distribution. Similarly, our lab successfully demonstrated the ability of CRAd-loaded NSCs to migrate to distant tumor sites and prolong mice survival in orthotopic models of human-derived glioblastoma (Ahmed et al., 2011a,b). Furthermore, Yong RL et al. have shown that MSCs loaded with Ad.Δ24-RGD administered by carotid artery injection in nude mice bearing human-derived glioma xenografts were able to cross the BBB and effectively reach the targeted tumor site (Yong et al., 2009). The researchers observed that loaded MSCs could also deliver their viral payload to the neoplastic area, leading to tumor eradication and improved animal survival. The above results highly suggest the potent tropism of stem cell carriers toward glioma. In addition to NSCs and MSCs, other cells have been used in pre-clinical studies as potential viral carriers. Some examples include endothelial cells (Iankov et al., 2007), cytokine-induced killer (CIK) cells (Thorne et al., 2006), dendritic cells (Ilett et al., 2009), monocytes (Iankov et al., 2007) and T-cells (Qiao et al., 2008; Ilett et al., 2009). Indeed, Qiao et al. have recently demonstrated that naïve T cells were able to efficiently deliver oncolytic viruses to lymphoid organs sheltering metastatic cells (Qiao et al., 2008). They further showed that the reduction of metastatic burden by viral oncolysis led to a posterior anti-tumor immune response with tumor eradication. Although mostly effective in the pre-clinical setting, each delivery vehicle has its own advantages and limitations. Stem cell carriers, however, are the only ones that are able to combine immunosuppressive and tumor-tropic properties, together with permissiveness for viral replication. Taken together, these characteristics make stem cell carriers excellent anti-tumor delivery vehicles, opening up new paths for targeted anti-cancer therapy.

### Limited tissue penetration and intratumoral distribution

Upon reaching the target site, oncolytic viruses need to accomplish several steps in order to achieve clinically relevant therapeutic efficacy. They must replicate, spread efficiently across the tumor interstitium and eradicate as many neoplastic cells as possible without disturbing the adjacent non-neoplastic tissue. However, the inability of the virus to spread beyond physical barriers inside the complex tumor microenvironment is responsible for prematurely compromising viral spread within the tumor bulk, limiting the success of the treatment. Surrounding connective tissue, extracellular matrix (ECM) and necrotic pockets of destroyed tumor tissue enclosed by ECM are considered the main factors accountable for an uneven intratumoral penetration and distribution of oncolytic viruses (Maillard et al., 1998). Based on this concept, Kuriyama et al. used proteolytic enzymes as a pretreatment of human-derived glioma xenografts implanted in mice. They were able to demonstrate that digestion of ECM significantly increased intratumor virus mediated-gene transduction (Kuriyama et al., 2000). Similarly, pretreatment of different tumor models with collagenases (McKee et al., 2006), human hyaluronidase enzyme (Ganesh et al., 2008) and matrix metalloproteinases (MMPs-1 and 8) (Brinckerhoff and Matrisian, 2002; Mok et al., 2007) was able to efficiently disrupt tumor-related ECM and increase anti-tumor efficacy of oncolytic viruses. Furthermore, genetically engineered viruses have also proven to be a nice tool for tumor ECM modulation. Kim and authors were able to create an oncolytic adenovirus expressing relaxin, a peptide responsible for inducing expression of MMPs. Their results showed that, compared to the unmodified virus control, transduction of subcutaneous tumors with Ad-DeltaE1B-RLX revealed increased intratumoral virus distribution and potent tumor regression (Kim et al., 2006; Ganesh et al., 2007). Another possible option to increase poor virus transduction imposed by physical barriers is the use of virus-loaded carrier cells. Such carriers are capable of migrating toward the tumor site, extravasating from blood vessels, and moving across the tumor microenvironment (Ahmed et al., 2011a). Thus, they can effectively deliver their viral payload to different intratumoral areas and successfully induce tumor regression. Taken together, the above results highlight the importance of establishing even virus distribution throughout the entire tumor tissue in order to obtain maximal therapeutic benefits.

### Strong host-mediated antivirus immunity and lack of long-lasting anti-tumor immune response

The anti-tumor therapeutic effect of oncolytic viruses comprises a subtle equilibrium between anti-viral and anti-tumor immune responses. Innate and adaptive immune systems are the two components responsible for defending the host from viral infections. While mediators of the innate immune system work as the first line of defense against pathogens, the adaptive immune system is the major player in long lasting immunity. In the context of oncolytic virotherapy, upon vector administration, mononuclear cells are rapidly recruited to the injection site (Fulci et al., 2007). In order to limit viral propagation, these first responders mobilize additional reinforcements through signaling for maturation of antigen presenting cells and activation of the adaptive immune response (Wakimoto et al., 2003). Consequently, the innate immune system is one of the major obstacles for achieving effective viral delivery and replication at the tumor site, impeding successful tumor destruction (Balachandran and Barber, 2004; Abordo-Adesida et al., 2005; Friedman et al., 2006). Otsuki et al. and Fulci et al. demonstrated that elimination of antiviral cytokines and depletion of mononuclear cells significantly increased intratumoral viral titers and led to important tumor regression (Fulci et al., 2007; Otsuki et al., 2008). Moreover, pretreatment of intracranial tumors with antiangiogenic agents prior to administration of oncolytic virotherapy has proven to reduce intratumoral infiltration of antiviral immune cells and increase viral propagation at the tumor site (Kurozumi et al., 2007). The combination of oncolytic virotherapy strategies and single or multiple immune suppressants is currently under investigation in preclinical studies. The main goal here is to inhibit both innate and adaptive immune responses, opening a free way for intratumoral viral transduction, propagation and spread (Fulci et al., 2006). Another option that has been extensively studied is the use of immunosuppressive stem-cell carriers to deliver oncolytic vectors to distant tumor sites, bypassing the anti-virus host immune response (Power et al., 2007; Ahmed et al., 2011a,b).

Although antiviral immunity has proven to be prejudicial for a successful oncolytic virotherapy, stimulation of the adaptive immune system holds a positive impact on such therapeutic approach due to the possibility of generation of a long-lasting anti-tumor immune response (Todo et al., 2001b). This vaccination effect is believed to happen following the activation of cytotoxic T lymphocytes (CTLs). CTLs recognize viral antigens presented on the surface of neoplastic cells and are redirected to tumor-specific antigens, leading to increased efficacy of the oncolytic virotherapy and anti-tumor immunity (Todo et al., 1999; Thomas and Fraser, 2003; Curtin et al., 2009). One important concept is that not all malignant cells within a tumor need to be targeted by oncolytic viruses in order to initiate an efficient anti-tumor immune response. Upon extermination of only few neoplastic cells, the process of tumor-specific antigen presentation starts. This activity may trigger potent anti-tumor immunity, which consequently increases the probability of effective tumor eradication (Qiao et al., 2008). Anti-tumor immune response can also be enhanced by the use of genetically engineered oncolytic viruses loaded with genes that encode for many cytokines. Researchers have recently shown that mice treated with vector constructs that were able to encode for interleukin-2 (IL-2) (Mizuno et al., 2000), IL-4 (Post et al., 2007), or IL-12 (Stanford et al., 2007) cytokines presented a rapid and sustained tumor regression.

However, therapeutic anti-tumor vaccination post oncolytic virotherapy administration is still a far reality. First, overcoming the strong anti-virus innate immune response for an efficient virus transduction is still a challenge. Therefore, even though oncolytic viruses manage to reach the tumor site and transduce cells, it is not clear that tumor-specific antigens will be presented to CTLs, which in turn will activate the anti-tumor adaptive immune system. Second, due to high intratumoral heterogeneity, CTLs that target just some few tumor antigens are less likely to be effective. And third, malignant glioma rapid progression may outpace the time necessary for adequate adaptive immune response activation. Thus, by the time that a fair activation is noticeable, the tumor is already uncontrollable, with marked clinical deterioration (Rosenberg et al., 2004). Only a better understanding of the mechanisms that dictate host immunity to oncolytic virotherapy will allow the generation of better alternatives to achieve a fine tune between host-antiviral and anti-tumoral immune responses, leading to better therapeutic outcomes. Additionally, since the CNS immune system presents unique properties, such as immune tolerance, which differentiates it from all the other organs, further investigations on tumor/viruses interactions in this field are still needed.

### Inaccuracy of current preclinical animal models

The currently available inaccurate preclinical animal models compose a significant obstacle to translating targeted virotherapy into effective treatments. Since animals typically do not spontaneously develop brain tumors, pre-clinical studies mostly depend on artificial systems to evaluate the efficacy of therapeutic approaches that will be further translated to humans. As a consequence, such models are usually not representative of the heterogeneous nature of human gliomas and do not faithfully recapitulate the complexity of tumor-host immunological interactions. An ideal glioma model should mimic the genetic and histological alterations seen in humans, present predictable growth and reproducible progression patterns, in addition to being non-immunogenic (Candolfi et al., 2007). As such model do not yet exist, the most appropriate glioma model should be selected depending on the aims of the proposed investigation.

Mouse models are the most widely used ones due to animal availability, easy breeding and well-known genetic profile. Two major strategies are currently available for generating gliomas in experimental models: direct implantation of glioma xenografts or spontaneous tumor formation in genetically engineered mice. Xenograft models are generated by implantation of cultured glioma cells of human or rodent origin in immunodeficient mice. The resulting tumors are known to be easily reproducible, with rapid formation and high penetrance (Shapiro et al., 1979). Nevertheless, such tumors have major disadvantages. First, they lack stepwise genetic changes that are characteristics of human gliomas. Second, there is an absence of host/tumor immunological interactions, which account for some false positives seen in pre-clinical trials. Last, many of these tumors lack accurate histological vascularization and hardly recapitulate the characteristics of the original tumor (Finkelstein et al., 1994). Although these xenograft models are widely used in therapeutic testing due to their practicality and reproducibility, they are not yet able to faithfully mimic spontaneous human gliomas. As a consequence, the reproduction of their exciting therapeutic outcomes in clinical trials is still unclear.

As an attempt to overcome the above issues, researchers developed a very attractive option: genetically engineered mouse models. In these animals, spontaneous tumor formation takes place due to specific mutations in genes that are responsible for glioma initiation and maintenance in humans. This model is especially interesting because it is capable of recapitulating the biological mechanisms present in human gliomas, exhibiting tumor-host interactions, reproducing tumor infiltration and stepwise genetic alterations, and identifying causative mutations and possible therapeutic targets (Pelengaris et al., 1999). Although the time-scale is very different from that of human patients, these slow-growing tumors provide a nice perception of how the evolving tumor may affect the host, in addition to delivering important insights onto the effectiveness of therapeutic agents on the preformed tumor (Fomchenko and Holland, 2006). However, when used in brain tumor research, genetically engineered mouse models present important drawbacks. First, solely depending on spontaneous tumor models for glioma investigation is problematic due to extended latency of tumor formation, low penetrance, difficult reproducibility and the requirement of sophisticated *in vivo* imaging systems. Second, the genetic settings of these spontaneous malignancies are relatively simple when compared to multiple and complex genetic abnormalities harbored by human-derived malignant gliomas. Third, particularly in these models, genetic alterations are present in the whole animal or tissue, while the specific mutations that are accountable for initiating human gliomas are more likely to arise from single cells or small transformed populations (Hu and Holland, 2005). Fourth, mouse models that bear spontaneous tumor formation are not able to support conditionally replicative oncolytic adenovirus replication, which poses an important limitation to the study of CRAd biodistribution and host anti-viral immune response. This issue could be tackled with the use of cotton rats for studies that focus on viral fate and immune interactions. However, most of the rat glioma models are derived from outbred animals, which result in a difficult interpretation of any given immunotherapeutic strategy. Genetically engineered models are ideal for investigating host/tumor immunity, proposing mechanisms for causative mutations and for identifying specific therapeutic targets, but crucial genetic and molecular differences from human gliomas still remain. Therefore, even if a specific therapeutic approach shows relative success in pre-clinical trials, there is no warranty that it will work as well in the clinical setting.

## Conclusion

Oncolytic virotherapy as a therapeutic approach for malignant glioma is still in its infancy. Although pre-clinical and phase I clinical trials have demonstrated its safety, more studies still need to be performed in order to fully characterize viral fate and to secure minimal neurotoxicity. Moreover, many trials have been made in small, selected and heterogeneous populations, which may mask the chances of a reliable prediction of a specific therapeutic intervention (Stanley, 2007). The absence of a vector system that allows for minimally invasive administration with high effectiveness, low intratumoral distribution post-vector administration, a strong anti-virus host immune response, the lack of a long lasting anti-tumor immunity and the inaccuracy of the currently available preclinical animal models are the major hurdles for translating this therapeutic approach into the clinical setting. The immune system plays a major role in dictating the efficacy of oncolytic viruses. Presently, there are two opposing lines of thoughts: one that defends the recruitment of the host-related immune response, and another that wishes its suppression. Both avenues have their benefits. On the one hand, genetically engineered oncolytic viruses armed with immunostimulatory agents have demonstrated important therapeutic effects. On the other hand, single or multiple therapeutic agents that are able to suppress the innate immune system have been shown to significantly increase intratumoral virus distribution and promote tumor regression. To prove which of these lines is more fruitful, new preclinical and clinical studies are still necessary. However, we believe that defining anti-virus and anti-tumor host immune responses is crucial for achieving maximal therapeutic efficacy.

Some important topics still need to be addressed before new or modified oncolytic viruses reach the clinical practice. First, all viruses tested in clinical trials should be closely monitored for replication or dissemination outside tumor areas. The patient's immune system should be carefully observed to allow a better understanding of how the host immunity reacts on the presence of these vectors. Second, newly generated oncolytic viruses must be completely safe and viral delivery needs to be optimized so physical and physiological barriers can be surpassed. Third, a combination of oncolytic virotherapy and conventional anti-glioma therapies should be employed in order to improve therapeutic efficacy. Last, any oncolytic virotherapy that aims to reach the clinical practice should be able to target CSCs. Since CSCs are believed to be a permanent reservoir of malignant glioma, eradicating these cells may prevent tumor recurrence. Although the translation of targeted virotherapies from preclinical models to the clinical practice is still full of challenges and limitations, pursuing this path is justified due to the rapid progress already achieved and the undeniable potential efficacy of this approach.

### Conflict of interest statement

The authors declare that the research was conducted in the absence of any commercial or financial relationships that could be construed as a potential conflict of interest.
